# Numerical analysis for temporal and spectral responses of electromagnetic waves in spatially homogeneous time varying medium

**DOI:** 10.1038/s41598-024-64874-z

**Published:** 2024-07-02

**Authors:** Ahmed M. Attiya, Eman M. Eldesouki

**Affiliations:** 1 Microwave Engineering Department, Electronics Research Institute (ERI) Cairo, Cairo Governorate, Egypt; 2 School of Sciences and Engineering, American University in Cairo (AUC), New Cairo, Egypt

**Keywords:** Ordinary differential equations, Time varying medium, Frequency domain response and metamaterial, Engineering, Mathematics and computing

## Abstract

This paper presents simple numerical solutions for electromagnetic plane waves in spatially homogenous time varying medium. The solution is based on converting the resulting second order differential equation into two combined ordinary differential equations which are solved numerically by using the built-in ode113 function in Matlab. By using this method, the time domain responses of the electric and magnetic fields at fixed point in space are obtained. The proposed method is applied on two cases: linearly time varying medium and sinusoidally time varying medium. The corresponding frequency domain response is obtained by using inverse Fourier transformation of the obtained time domain response. The proposed method is compared with FDTD solution. It is found that the proposed method has the same accuracy of FDTD with much less computational time.

## Introduction

The study of electromagnetic wave interactions with time varying media is an important area of research in many practical applications like telecommunications^1^, space plasma^2^, terahertz technology^3^, metamaterials^4^, nonreciprocal systems^5^, photonics^6^, and non-linear optics^7^. Understanding the behavior of electromagnetic waves in such media is essential for the design and analysis of these systems that involve time-varying electromagnetic properties, such as materials with changing permittivity, permeability, or conductivity. A pioneering research in the field of time-varying media can be attributed to Morgenthaler who studied electromagnetic wave propagation in a medium with a time modulated parameters^8^. It was demonstrated that reflection and transmission of electromagnetic waves are not only due to spatial inhomogeneities, but also due to temporal change of the material parameters. After that, many interesting and novel phenomena were discovered, such as interband photonic transitions^9^, efficient frequency conversion^10^, dispersion^11^, Fresnel drag^12^ and nonreciprocal gain^13^. In many practical situations, the rate of changing the medium properties with time are much smaller than the characteristic frequency of the electromagnetic waves under consideration such as wave propagation through inhomogeneous dielectric media^14^ or Metamaterials^15^. This slow variation allows for the application of the slowly varying approximation that describe wave propagation and radiation characteristics of the system.

The behavior of electromagnetic plane waves in spatially homogeneous time-varying media is described by Maxwell's equations. The solutions of these equations depend on the boundary conditions at the interfaces between different media, as well as the properties of the media themselves. To calculate the fields in such media, several analytical or numerical methods can be employed, depending on the specific characteristics and complexity of the problem. Analytical solutions were driven for homogeneous media^16,17^, the polarizability of single particles^18,19^ or planar structures^20–22^. These methods are obtained by solving the Maxwell's equations in partial differential form using mathematical techniques such as separation of variables, Fourier series, or Laplace transforms^23^. Many of these analytical solutions use conversion matrix technique to analyze harmonic generation^24^. On the other hand, numerical methods are useful when dealing with complex scenarios. One widely used numerical method is Finite-Difference Time-Domain (FDTD) method, which discretizes both space and time domains into a grid and solves the time-domain Maxwell's equations in discretized steps^25^. However, FDTD requires significant computational resources and may become computationally expensive for large-scale problems.

The present paper introduces a simple and fast approach for solving the differential form of Maxwell’s equations in a time-varying medium. This method is used here to study the effects of different modulation schemes of different time varying media on the response of propagating waves inside these media. The proposed method is based on using the Matlab built-in function of ordinary differential equation (ode113). The novelty of our work lies in the unique combination of the wave equations, the time-varying medium, and the specific numerical solver employed. Our paper aims to provide a practical and accessible solution for researchers and engineers working with electromagnetic waves in time-varying media. The ode113 function is based on Adams Method for solving nonstiff ordinary differential equations. For the present problem, Maxwell’s equations are defined as a system of second-order differential equations. These second-order differential equations can be decomposed into a coupled system of first order differential equations. These first order differential equations are solved numerically by using ode113 function to obtain the time domain response of electromagnetic waves inside the time varying medium. This function adjusts the time step size dynamically, providing high accuracy, efficient use of computational resources and ensures that the solution captures important features of the time-domain response accurately.

To validate the effectiveness of the proposed method, it is applied on two specific cases: linearly time-varying media and sinusoidally time-varying media. Specifically, the effects of simultaneously varying both the permittivity and permeability, as well as the effects of varying the permittivity alone are investigated. An example of a time-dependent material with permittivity and permeability varying linearly with time is Vanadium Dioxide (VO_2_) thin film. Vanadium dioxide is an attractive material that exhibits a metal–insulator transition (MIT) at a critical temperature (around 68 °C). However, this transition can also be triggered by an external stimulus (light irradiation). At room temperature, VO_2_ behaves as a semiconductor with relatively low permittivity and permeability (ε_1_ and μ_1_). When exposed to a strong light pulse, VO_2_ undergoes a rapid transition to a metallic state. During this transition phase, the permittivity and permeability can be approximated as linearly increasing with time (t) as the material properties change. The slope of the linear variation ($d\varepsilon /dt$ and $d\mu /dt$) represents the rate at which the permittivity and permeability increase with time. This rate depends on the intensity and duration of the light pulse. Once the light pulse ceases, VO_2_ might gradually cool down and return to its initial semiconducting state with ${\varepsilon }_{1}$ and ${\mu }_{1}$. The return transition could also be modeled linearly, but with a negative slope. During the rapid transition phase triggered by the light pulse, a linear approximation can be a reasonable first-order model to capture the essence of the changing material properties. Understanding the interaction of electromagnetic waves with VO_2_ during its light-induced transition can be relevant for developing optical switches or modulators. By controlling the light pulse characteristics, we can potentially design materials that exhibit a desired rate of change in permittivity and permeability, leading to specific wave manipulation functionalities^26^. On the other hand, an example of a time-dependent material with permittivity and permeability varying sinusoidally with time is a tunable metamaterial with electrically reconfigurable properties. The key aspect is that the metamaterial's properties can be dynamically controlled using external stimuli like an electric field. Both permittivity and permeability are chosen to have a base value (${\varepsilon }_{ro}$ and ${\mu }_{ro}$, respectively) representing the material's intrinsic properties. These base values are then modulated by a sinusoidal function of time (t) with a specific amplitude ($\Delta \varepsilon$ and $\Delta \mu$) and frequency ($\omega$). The amplitude ($\Delta \varepsilon$ and $\Delta \mu$) of the sinusoidal variations can be controlled by applying an external electric field. By changing the strength of the electric field, we can adjust the degree to which the permittivity and permeability deviate from their base values. This allows for dynamic tuning of the metamaterial's response to electromagnetic waves. Such a metamaterial could be used to create tunable filters that can selectively block or transmit specific frequencies based on the applied electric field^27^. It could also be used for dynamically adjusting the phase and amplitude of electromagnetic waves, potentially leading to applications in beam steering or cloaking devices^28,29^. By analyzing the time-domain responses, it would be possible to gain insights into the behavior of electromagnetic waves in these media and their temporal variations. This time domain response is then converted to frequency domain via Fourier transform to obtain also the corresponding spectral response.

The organization of the remaining parts in the present paper are as follows: Section "Formulation of the problem" presents the formulation of the problem of electromagnetic wave propagation in time varying medium. Section "Numerical solution of second order differential equation" presents an example for solving second order differential equation by using Matlab function ode113 and comparing the numerical solution with the corresponding analytical solution to show the accuracy of the proposed method. Section "Numerical solution of electromagnetic waves in time varying medium" presents numerical solutions for wave propagation in time varying media for different cases. Finally, Section "Conclusion" presents concluding remarks.

## Formulation of the problem

**Figure 1:**
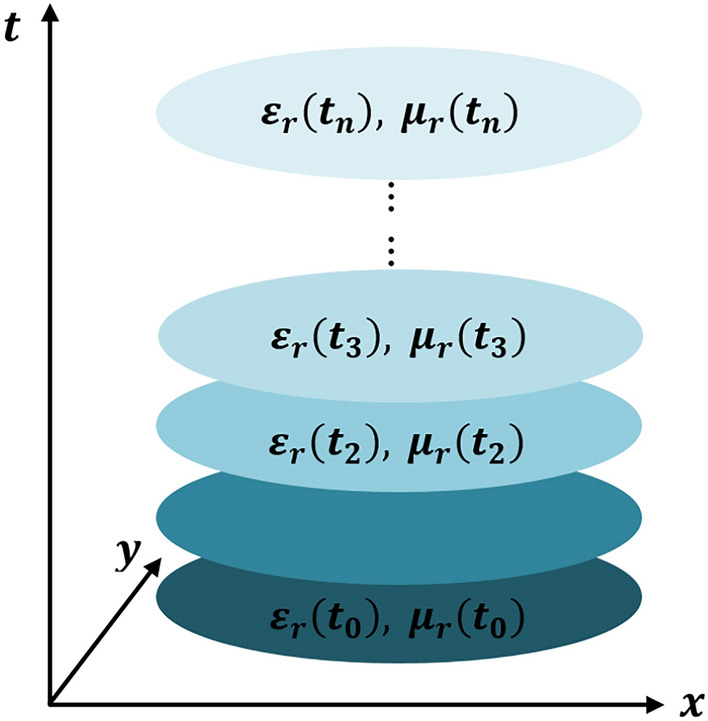
Schematic representation of time-varying medium.

Figure 1 shows a schematic representation of time varying medium. Electromagnetic waves in source free time varying medium can be represented as:

1a $$\nabla \times \text{E}=-\frac{\partial \mu \text{H}}{\partial t}$$

1b $$\nabla \times \text{H}=\frac{\partial \varepsilon \text{E}}{\partial t}$$

where $={\varepsilon }_{o}{\varepsilon }_{r}(t)$ , $\mu ={\mu }_{o}{\mu }_{r}(t)$ are the time varying permittivity and permeability respectively.

The wave equation of electric field in this case is given by:

2 $${\nabla }^{2}\text{E}=\frac{\partial \mu }{\partial t}\frac{\partial \varepsilon }{\partial t}\text{E}+\varepsilon \frac{\partial \mu }{\partial t}\frac{\partial \text{E}}{\partial t}+2\mu \frac{\partial \varepsilon }{\partial t}\frac{\partial \text{E}}{\partial t}+\mu \varepsilon \frac{{\partial }^{2}\text{E}}{\partial {t}^{2}}+\mu \frac{{\partial }^{2}\varepsilon }{\partial {t}^{2}}\text{E}$$

A similar wave equation can be obtained for the magnetic field. Assuming that the electric field has only *y* component and it is propagating in *z* direction, the above equation can be simplified as follows:

3 $$\frac{{\partial }^{2}{E}_{y}}{\partial {z}^{2}}=\mu \varepsilon \frac{{\partial }^{2}{E}_{y}}{\partial {t}^{2}}+\left(\varepsilon \frac{\partial \mu }{\partial t}+2\mu \frac{\partial \varepsilon }{\partial t}\right)\frac{\partial {E}_{y}}{\partial t}+\left(\mu \frac{{\partial }^{2}\varepsilon }{\partial {t}^{2}}+\frac{\partial \varepsilon }{\partial t}\frac{\partial \mu }{\partial t}\right){E}_{y}$$

The solution of this wave equation can be represented by separation of variables as follows:

4 $${E}_{y}(z,t)=\frac{1}{\sqrt{\mu }\varepsilon }\Lambda (z){W}_{E}\left(t\right)$$

where $\Lambda (z)$ can be represented in terms of forward and backward propagating waves as follows:

5a $$\Lambda \left(z\right)=A{e}^{-j\beta z}+B{e}^{+j\beta z}$$

Thus the spatial dependence of $\Lambda \left(z\right)$ can be represented in a differential form as follows:

5b $$\frac{1}{\Lambda (z)}\frac{{\partial }^{2}\Lambda (z)}{\partial {z}^{2}}=-{\beta }^{2}$$

Thus, the second-order derivative of the electric field component, ${E}_{y}$, in terms of its spatial variation along the $z$-axis is related to the propagation constant $\beta$ is given by:

6 $$\frac{{\partial }^{2}{E}_{y}}{\partial {z}^{2}}=-{\beta }^{2}{E}_{y}$$

Thus, the corresponding temporal wave equation is given by::

7 $$\frac{{\partial }^{2}{W}_{E}}{\partial {t}^{2}}-\frac{1}{2\mu }\frac{{\partial }^{2}\mu }{\partial {t}^{2}}{W}_{E}+\frac{1}{4{\mu }^{2}}{\left(\frac{\partial \mu }{\partial t}\right)}^{2}{W}_{E}+\frac{{\beta }^{2}}{\mu \varepsilon }{W}_{E}=0$$

where

8a $${\beta }^{2}={\omega }^{2}{\mu }_{0}{\mu }_{r0}{\varepsilon }_{0}{\varepsilon }_{r0}$$

8b $${\mu }_{r0}={\mu }_{r}(t=0)$$

8c $${\varepsilon }_{r0}={\varepsilon }_{r}(t=0)$$

For time-varying medium, ${\mu }_{r}(t)$ and ${\varepsilon }_{r}(t)$ are functions of time as follows:

8d $$\frac{{\beta }^{2}}{\mu \varepsilon }={\omega }^{2}\frac{{\mu }_{r0}{\varepsilon }_{r0}}{{\mu }_{r}(t){\varepsilon }_{r}(t)}$$

8e $$\omega =2\pi f$$

where $f$ is the operating frequency of electromagnetic wave.

By using similar steps, the magnetic field can be written in space and time as:

9 $${H}_{x}(z,t)=\frac{1}{\sqrt{\varepsilon }\mu }{W}_{H}\left(t\right)\Lambda (z)$$

and

10 $$\frac{{\partial }^{2}{W}_{H}}{\partial {t}^{2}}-\frac{1}{2\varepsilon }\frac{{\partial }^{2}\varepsilon }{\partial {t}^{2}}{W}_{H}+\frac{1}{4{\varepsilon }^{2}}{\left(\frac{\partial \varepsilon }{\partial t}\right)}^{2}{W}_{H}+\frac{{\beta }^{2}}{\mu \varepsilon }{W}_{H}=0$$

The two wave Eqs. (7) and (10) describe electromagnetic waves in homogeneous isotropic time varying media for both electric and magnetic fields. However, it is difficult to find exact analytical solutions for these two equations. Assuming that the rates of changing $\varepsilon$ and $\mu$ with time are much smaller than the frequency of electromagnetic waves, these wave equations correspond to slowly varying second-order differential equations (SV-SODE)^30^. Thus, to find electric and magnetic fields inside a time modulated medium, it is required to solve these two SV-SODEs. In the following section, an efficient technique to solve SODE by using a built-in Matlab function is demonstrated for a known SODE and verified by comparing with its analytical solution. After that, the built-in Matlab function is used to obtain the solutions of the developed wave equations of time modulated media.

## Numerical solution of second order differential equation

The wave equations in (7) and (10) can be presented in a general form of a second order differential equation as follows:

11 $$-\frac{{\partial }^{2}y}{\partial {t}^{2}}+Q\left(t\right)y={\lambda }^{2}y$$

where, $y$ is an unknown function of time, $\lambda$ is a constant, $Q\left(t\right)$ is a time-varying coefficient. By introducing an intermediate variable ${y}_{2}=\partial y/\partial t$, (11) can be reformulated as a first order deferential equation as follows:

12 $$\frac{\partial {y}_{2}}{\partial t}=Q\left(t\right)y-{\lambda }^{2}y$$

This system of coupled first order differential equation can be solved by using the ode113 Matlab function. The inputs of ode113 function are the set of differential equations to be solved, their initial conditions, and the time interval. The outputs of ode113 function are vectors of time points and the corresponding values of the state variables; $y$ and ${y}_{2}$.

As an example for a second order differential equation which can be presented by (11) is:

13a $$4{\left({t}^{2}+1\right)}^{2}\frac{{\partial }^{2}y}{\partial {t}^{2}}+\left(a{t}^{2}+a-3\right)y=0$$

The analytical solution of this equation is given by^26^:

13b $$y={\left({t}^{2}+1\right)}^{1/4}\left({C}_{1}\text{cos}\xi +{C}_{2}\text{sin}\xi \right), \text{if} a>1$$

where:

13c $$\xi =\frac{1}{2}\sqrt{a-1}\text{ln}\left(t+\sqrt{{t}^{2}+1}\right)$$

Assuming that the initial conditions of $y\left(0\right)=0$ and ${\left.\frac{\partial y}{\partial t}\right|}_{t=0}=0.5$ with $a=2$, thus, (13a–13c) can be rewritten as:

14a $$\frac{{\partial }^{2}y}{\partial {t}^{2}}+\frac{\left(2{t}^{2}-1\right)}{4{\left({t}^{2}+1\right)}^{2}}y=0$$

and the corresponding analytical solution is given by:

14b $$y={\left({t}^{2}+1\right)}^{1/4}\text{sin}\left(\frac{1}{2}\text{ln}\left(t+\sqrt{{t}^{2}+1}\right)\right)$$

14c $$\xi =\frac{1}{2}\text{ln}\left(t+\sqrt{{t}^{2}+1}\right)$$

**Figure 2:**
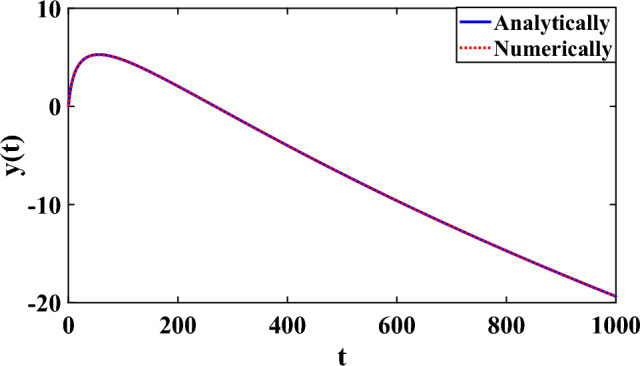
Comparison between numerical and analytical solutions of the 2nd order D.E.

Figure 2 shows a comparison between the numerical solution of (14a) by using ode113 and the exact analytical solution. It can be noted the excellent agreement between the obtained numerical solution and the analytical solution. This result can be considered as a good indication that ode113 is efficient function to solve SV-SODE.

## Numerical solution of electromagnetic waves in time varying medium

The proposed numerical method mentioned in Section "Numerical solution of second order differential equation" is applied in this Section to investigate the time-domain response of electromagnetic plane waves in time varying media. Two specific cases are discussed, linearly time-varying media and sinusoidally time-varying media. The first case can be suitable for linearly scanning antenna applications. On the other hand, the second case can be suitable for frequency mixing and harmonic generation applications. The effects of varying the permittivity and permeability simultaneously, as well as the effects of varying the permittivity only are investigated. These effects are investigated on the amplitude of electric and magnetic fields and the instantaneous frequency at an arbitrary observation point in space.

Furthermore, the obtained electric and magnetic fields obtained by the proposed numerical method are compared to the results of FDTD for comparison. The update equations of electric and magnetic fields for the 1D-FDTD in this case are given by^31^:

15a $$ E_{y}^{{n + \frac{1}{2}}} \left( i \right) = \frac{{\varepsilon ^{{n - \frac{1}{2}}} \left( i \right)}}{{\varepsilon ^{{n + \frac{1}{2}}} \left( i \right)}}E_{y}^{{n - \frac{1}{2}}} \left( i \right) + \left( {\frac{{\Delta t}}{{\varepsilon ^{{n + \frac{1}{2}}} \left( i \right)}}} \right)\left( {\frac{{H_{x}^{n} \left( {i + 1/2} \right) - H_{x}^{n} \left( {i - 1/2} \right)}}{{\Delta z}}} \right) $$

15b $$H_{x}^{{n + 1}} {\text{(}}i + 1/2) = H_{x}^{n} {\text{(}}i - 1/2) + \left( {\frac{{\Delta t}}{{\mu ^{{n + 1}} (i)}}} \right)\left( {\frac{{E_{y}^{{n + \frac{1}{2}}} \left( {i + 1} \right) - E_{y}^{{n - \frac{1}{2}}} \left( i \right)}}{{\Delta z}}} \right)$$

where $\Delta t$ is the time step and $\Delta x$ is the cell size (15b).

### Linearly-time varying medium

Assume that both permittivity and permeability of the medium are varying linearly with time as follows:

16a $${\varepsilon }_{r}={\varepsilon }_{r0}\left(1+at\right)$$

16b $${\mu }_{r}={\mu }_{r0}\left(1+bt\right)$$

where, the initial relative permittivity and permeability at $t = 0$ are ${\varepsilon }_{r0}=1$ and ${\mu }_{r0}=1$, respectively. $a$ and $b$ are constants that determine the rate of change of the permittivity and permeability with time. By applying (16a, 16b) the second-order partial differential equation of wave equations in (7) and (10) can be simplified as:

17a $$\frac{{\partial }^{2}{W}_{E}}{\partial {t}^{2}}+\frac{{b}^{2}}{4{\left(1+bt\right)}^{2}}{W}_{E}+{\omega }^{2}\frac{1}{\left(1+at\right)\left(1+bt\right)}{W}_{E}=0$$

17b $$\frac{{\partial }^{2}{W}_{H}}{\partial {t}^{2}}+\frac{{a}^{2}}{4{\left(1+at\right)}^{2}}{W}_{H}+{\omega }^{2}\frac{1}{\left(1+at\right)\left(1+bt\right)}{W}_{H}=0$$

This linearly varying medium is studied for two special cases. The first case where $\left(a=b\right)$.and the second case where $\left(b=0\right)$.

For the first special case, the rate of changing the permittivity and permeability with time is equal. By applying $\left(a=b\right)$, Eqs. (17a, 17b) can be simplified to:

18a $$\frac{{\partial }^{2}{W}_{E}}{\partial {t}^{2}}+\frac{{a}^{2}+4{\omega }^{2}}{4{\left(1+at\right)}^{2}}{W}_{E}=0$$

18b $$\frac{{\partial }^{2}{W}_{H}}{\partial {t}^{2}}+\frac{{a}^{2}+4{\omega }^{2}}{4{\left(1+at\right)}^{2}}{W}_{H}=0$$

where the time domain responses of the electric $$\left({E}_{y}\right)$$ and magnetic ($${H}_{x})$$ fields at a fixed point in space are obtained as:

19a $${E}_{y}(z=0,t)=\frac{1}{\sqrt{\mu }\varepsilon }{W}_{E}\left(t\right)$$

19b $${H}_{x}(z=0,t)=\frac{1}{\sqrt{\varepsilon }\mu }{W}_{H}\left(t\right)$$

**Figure 3:**
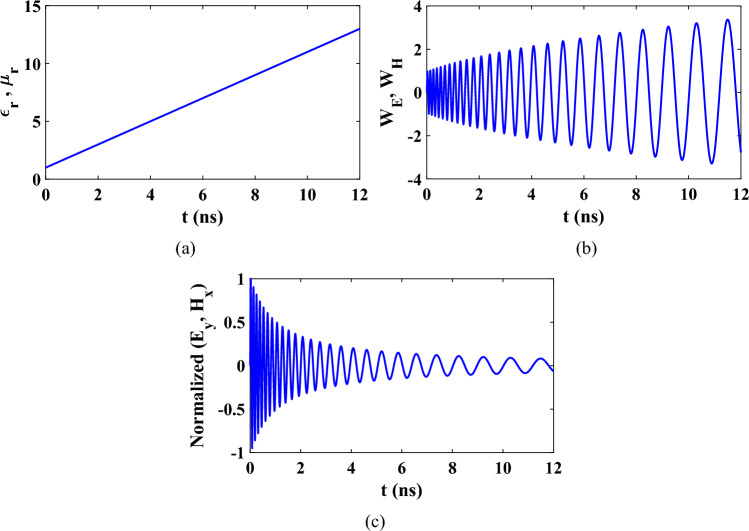
Time domain responses of (**a**) ${\varepsilon }_{r}$,${\mu }_{r}$ (**b**) ${W}_{E}$, ${W}_{H}$ and (**c**) Normalized electric and magnetic fields at fixed point in space for linearly time varying medium.

Assuming that operating frequency ${f}_{0}=10 \text{GHz}$ and the slowly varying parameters $a=b=0.1{f}_{0}$. The time domain response of the slowly varying $\varepsilon$ and $\mu$ is shown in Fig. 3a. The time domain response of ${W}_{E}$ and ${W}_{H}$ related to the electric and magnetic fields are obtained by solving (18a, 18b) by using ode113 function as shown in Fig. 3b. The corresponding electric and magnetic fields are shown Fig. 3c. From this figure, it can be noted that increasing both the permittivity and permeability simultaneously increase ${W}_{E}$ and ${W}_{H}$ and decrease the amplitude of electric and magnetic fields at the observation point in the space.

In the second case, the rate of changing the permittivity with time is only considered and the permeability of the medium is invariant with time $(b=0)$. Thus, (17a, 17b) can be simplified as follows:

20a $$\frac{{\partial }^{2}{W}_{E}}{\partial {t}^{2}}+{\omega }^{2}\frac{1}{\left(1+at\right)}{W}_{E}=0$$

20b $$\frac{{\partial }^{2}{W}_{H}}{\partial {t}^{2}}+\frac{{a}^{2}}{4{\left(1+at\right)}^{2}}{W}_{H}+{\omega }^{2}\frac{1}{\left(1+at\right)}{W}_{H}=0$$

**Figure 4:**
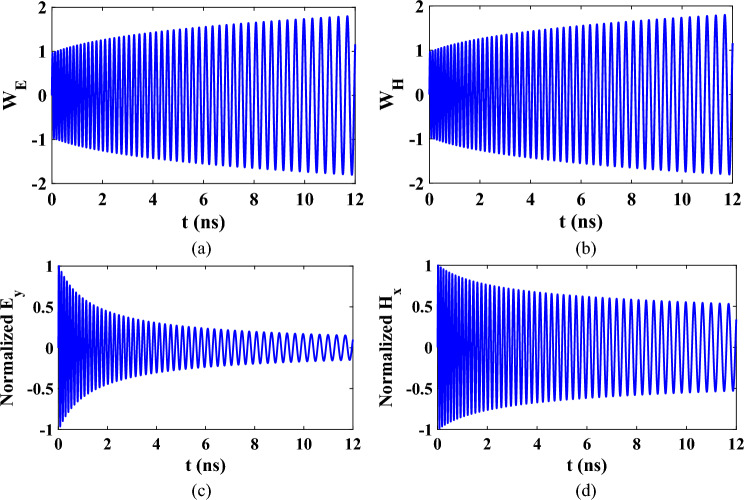
Time domain responses of (**a**) ${W}_{E}$, (**b**) ${W}_{H}$, (**c**) Normalized ${E}_{y}$, and (**d**) Normalized ${H}_{x}$ at fixed point in space for linearly time varying medium at ${f}_{0}=10\text{ GHz}$ and $a=0.1{f}_{0}$.

The time domain response of ${W}_{E}$ and ${W}_{H}$ related to the electric and magnetic fields are shown in Fig. 4a,b, respectively. The corresponding electric and magnetic field are shown Fig. 4c,d, respectively. From these figures, it can be noted that increasing the permittivity only would decrease the magnitude of the electric field with a higher rate compared to the decrease of the magnetic field.

It can be noted that increasing both the permittivity and permeability simultaneously decreases the instantaneous frequency at the observation point in the space and decreases the amplitude of electric and magnetic fields. On the other hand, increasing the permittivity only would decrease the magnitude of the electric field with a higher rate compared to the decrease of the magnetic field. This can be explained as follows: when both permittivity and permeability increase with the same rate the refractive index increases while the characteristic impedance is maintained constant. This constant characteristic impedance causes that the normalized response of the electric and magnetic fields would be the same. On the other hand, increasing the refractive index would decrease the instantaneous frequency and the instantaneous power density. Decreasing the instantaneous power density in this case would cause decreasing both electric and magnetic fields with the same rate. For the second case where the permittivity increases while the permeability is maintained constant, the instantaneous refractive index increases but the instantaneous characteristic impedance decreases. Thus the instantaneous frequency decreases but the magnitude of the instantaneous electric field decays more rapidly than the corresponding magnetic field due to the instantaneous decrease of the characteristic impedance.

### Sinusoidally-time varying medium

Another case for time varying medium, if both permittivity and permeability of the medium varied sinusoidally with time according to the following equations:

21a $${\varepsilon }_{r}\left(t\right)={\varepsilon }_{r0}\left(1+p\text{sin}\left({\omega }_{m}t\right)\right)$$

21b $${\mu }_{r}\left(t\right)={\mu }_{r0}\left(1+q\text{sin}\left({\omega }_{m}t\right)\right)$$

where, ${\varepsilon }_{r0}$ and ${\mu }_{r0}$ are initial relative permittivity and permeability at $t = 0$, $p$ and $q$ are the amplitudes of the sinusoidal variations of permittivity and permeability functions and ${\omega }_{m}$ is the angular frequency of the sinusoidal variation, and $t$ represents time. By following the same procedures applied in case 1, two second-order partial differential equations are obtained for wave propagating in sinusoidally time-varying medium as given by:

22a $$\frac{{\partial }^{2}{W}_{E}}{\partial {t}^{2}}+\frac{{\omega }_{m}^{2}q\text{sin}\left({\omega }_{m}t\right)}{2\left(1+q\text{sin}\left({\omega }_{m}t\right)\right)}{W}_{E}+\frac{1}{4}{\left(\frac{{\omega }_{m}q\text{cos}\left({\omega }_{m}t\right)}{\left(1+q\text{sin}\left({\omega }_{m}t\right)\right)}\right)}^{2}{W}_{E}+\frac{{\omega }^{2}}{\left(1+q\text{sin}\left({\omega }_{m}t\right)\right)\left(1+p\text{sin}\left({\omega }_{m}t\right)\right)}{W}_{E}=0$$

22b $$\frac{{\partial }^{2}{W}_{H}}{\partial {t}^{2}}+\frac{{\omega }_{m}^{2}p\text{sin}\left({\omega }_{m}t\right)}{2\left(1+p\text{sin}\left({\omega }_{m}t\right)\right)}{W}_{H}+\frac{1}{4}{\left(\frac{{\omega }_{m}p\text{cos}\left({\omega }_{m}t\right)}{\left(1+p\text{sin}\left({\omega }_{m}t\right)\right)}\right)}^{2}{W}_{H}+\frac{{\omega }^{2}}{\left(1+q\text{sin}\left({\omega }_{m}t\right)\right)\left(1+p\text{sin}\left({\omega }_{m}t\right)\right)}{W}_{H}=0$$

**Figure 5:**
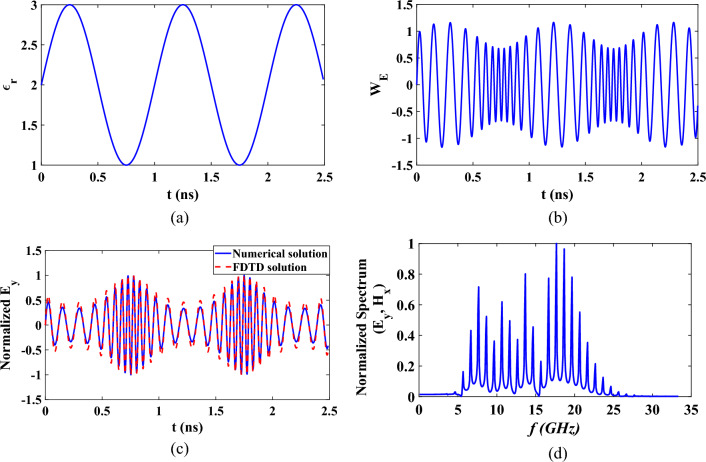
Time domain responses of (**a**) variation of ${\epsilon }_{r}={\mu }_{r}$, (**b**) ${W}_{E}={W}_{M}$, (**c**) Normalized electric field, and (**d**) Normalized spectrum of electric field at fixed point in space for sinusoidally varying medium.

Assuming that the permittivity and permeability of the medium are changed sinusoidally with the same rate such that $p=q=0.5$ with a modulation frequency ${f}_{m}=1 \text{GHz}$ and operating frequency $f=10 \text{GHz}$. The time varying $\varepsilon$ and $\mu$ are shown in Fig. 5a, where ${\varepsilon }_{r0}={\mu }_{r0}=2$. The corresponding time domain response of ${W}_{E}$ is shown in Fig. 5b. The corresponding electric field is shown Fig. 5c in comparison with the FDTD result. The FDTD results are obtained for an electrically thin slab of thickness 3 mm with spatial length of the cell $\Delta x=1.5 mm$ and $\Delta t=50 ns$. Good agreement between the result by the proposed numerical solution and the FDTD result. However, the computational time of the FDTD solution was nearly 50 times (40.05 s) the computational time for the same problem by using the proposed technique which is 0.801 s. The spectral response of this electric field is obtained by applying Fourier transformation as shown Fig. 5d. It is found that the temporal and spectral responses for both the electric and magnetic field are identical for this case.

**Figure 6:**
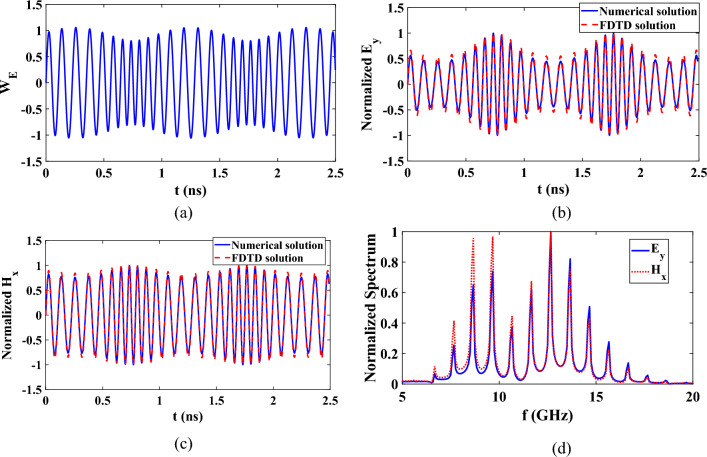
Time domain responses of (**a**) ${W}_{E}$, (**b**) Normalized electric field, (**c**) Normalized magnetic field, and (**d**) Normalized spectrum at fixed point in space for sinusoidally varying medium with ${\varepsilon }_{r0}=2$, ${\mu }_{r0}=1$.

For another case where the permittivity is with time is sinusoidally varying with time and constant permeability, $p=0.5$ and $q=0$. The time domain response of ${W}_{E}$ related to the electric field, is shown in Fig. 6a. The corresponding normalized electric and magnetic fields are shown in Fig. 6b,c. The spectral responses are also presented at Fig. 6d. It can be observed that these spectral responses of electric and magnetic fields are not the same.

The time and spectral responses of sinusoidally time varying medium can also be explained in a similar way to the response of increasing the permittivity and permeability. When both permittivity and permeability vary with the same response simultaneously, the time and spectral response of the normalized electric and magnetic fields would be identical. On the other hand, when only the permittivity is sinusoidally time varying, the instantaneous magnitude of the normalized electric field at the peak of the permittivity is more decreased than the corresponding magnetic field component. In addition, at the peak of the permittivity the instantaneous is decreased. This explains why the spectrum of low frequency components in Fig. 6c has greater magnetic field than electric field while at the high frequency components has nearly equal magnitudes for both electric and magnetic fields.

## Conclusion

In this paper a simple numerical technique is presented to solve the wave equations of electromagnetic plane waves in homogenous time varying medium. The solution is based on converting the second order differential equation of the corresponding wave equation into two coupled ordinary differential equations and solving these ordinary differential equations by using the built-in ode113 function in Matlab. This method is applied to obtain the time domain response of electromagnetic plane wave in linearly and sinusoidally time varying media. For both cases, we studied the effect of varying both the permittivity and permeability simultaneously. Also, the effect of varying the permittivity only is investigated. It is noted that increasing both the permittivity and permeability simultaneously decreases the instantaneous frequency at the observation point in the space and decreases the amplitude of electric and magnetic fields. On the other hand, increasing the permittivity only would decrease the magnitude of the electric field with a higher rate compared to the decrease of the magnetic field. The spectral response is also presented for the case of sinusoidally time varying media. It is found that the spectral responses for both the electric and magnetic field are identical for the case varying both the permittivity and permeability with the same rate. However, these spectral responses are different for case of varying the permittivity only. More extra results can be obtained by using this method for other different cases. These results can introduce significant insights for responses of electromagnetic waves in time varying media. These insights can be useful for developing applications for electromagnetic waves in time varying medium.

## Data Availability

The datasets used and/or analyzed during the current study available from the corresponding author on reasonable request.
